# Novel Modes of Extracting Teeth

**Published:** 1848-07

**Authors:** 


From the Dental Mirror and Brooklyn Annual Visiter.

The following gossip of our own is quite as rational as many of the remedies so highly puffed as cures for toothache:

### NOVEL MODE OF EXTRACTING TEETH.

Cover the tooth with hot ashes; bathe the gums for hours with boiling water; apply to the surrounding teeth and gums, "Sherman's Poor man's Plaster," leaving the crown of the tooth bare, when it will immediately *pass out!*"

### ANOTHER.

Bathe the parts as above for four hours and a half—pass into the nostrils by means of an air-pump, half an ounce of Scotch snuff. Keep the mouth distended until the tooth passes off by sneezing.

